# What Solder Can Do

**Published:** 1895-10

**Authors:** 


### What Solder Can Do.

"There are those," says *The Scientific Machinist*, Cleveland, August 1, "who have a natural taking to solder, and claim that they can do anything with this low-heat metal. Give them any substance and they are ready to go to work immediately. A glass water-gage was given to one of this fraternity, with a request to solder it into a brass elbow at both ends. The glass tube was heated to a red heat and covered with chlorid of silver till a reaction was formed and the surface of the glass well silverized; then a special solder was made to take hold of both the glass tube and the boiler fittings, and now there is a water column that will stand all that the boiler can stand, with no danger of its giving out in the packing."

